# Supplementary material for: Abnormal degradation of the neuronal stress-protective transcription factor HSF1 in Huntington's disease
Source: Nat Commun. 2017 Feb 13;8:14405. doi: 10.1038/ncomms14405 (PMC5316841; doi:10.1038/ncomms14405)
Supplement: Supplementary Information — Supplementary Figures and Supplementary Table [file ncomms14405-s1.pdf]

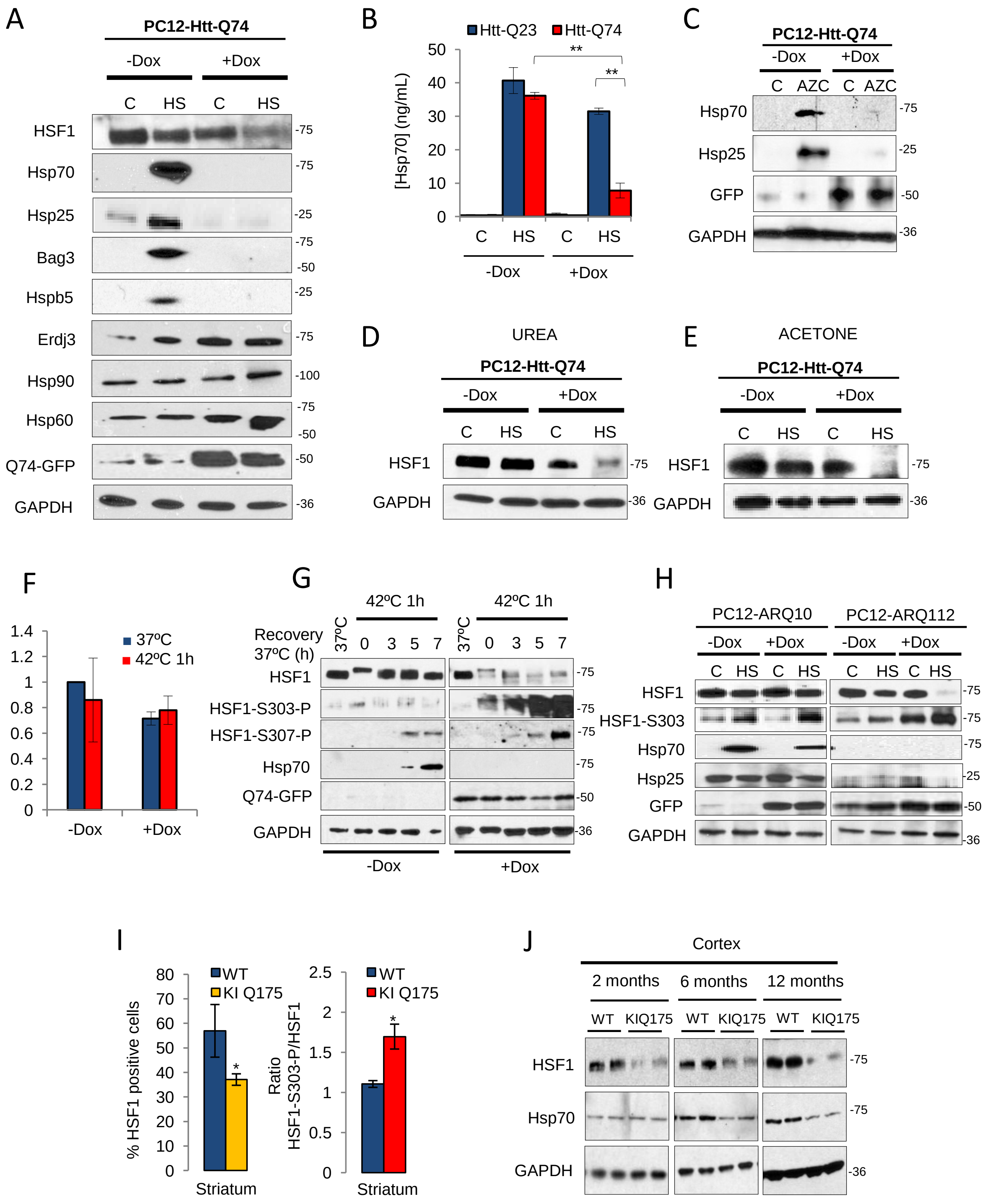
A
PC12-Htt-Q74
-Dox
+Dox
C
HS
C
HS
HSF1
Hsp70
Hsp25
Bag3
Hspb5
Erdj3
Hsp90
Hsp60
Q74-GFP
GAPDH
B
Htt-Q23
Htt-Q74
[Hsp70] (ng/mL)
**
**
C
HS
C
HS
-Dox
+Dox
C
PC12-Htt-Q74
-Dox
+Dox
C
AZC
C
AZC
Hsp70
Hsp25
GFP
GAPDH
D
UREA
PC12-Htt-Q74
HSF1
GAPDH
E
ACETONE
PC12-Htt-Q74
HSF1
GAPDH
F
HSF1 mRNA relative levels
37ºC
42ºC 1h
-Dox
+Dox
G
42ºC 1h
Recovery 37ºC (h)
37ºC
0
3
5
7
HSF1
HSF1-S303-P
HSF1-S307-P
Hsp70
Q74-GFP
GAPDH
-Dox
+Dox
H
PC12-ARQ10
PC12-ARQ112
HSF1
HSF1-S303
Hsp70
Hsp25
GFP
GAPDH
I
% HSF1 positive cells
WT
KI Q175
Striatum
Ratio HSF1-S303-P/HSF1
WT
KI Q175
Striatum
J
Cortex
2 months
6 months
12 months
WT
KIQ175
HSF1
Hsp70
GAPDH

Supplementary Figure 1. **HSF1 protein depletion in HD correlates with increased HSF1-S303/307 phosphorylation.** Related to Figure 1. (A) Htt-Q74 cells were cultivated at 37ºC (C) in the absence (-Dox) or presence of Doxycycline (+Dox) for 3 days and exposed to heat shock 1 h at 42ºC, followed by a recovery period at 37ºC for 7 h (HS). Protein samples were subjected to immunoblotting and the indicated proteins were analyzed using GAPDH as loading control. (B) Hsp70 protein levels were measured by ELISA (Enzo) under the same experimental conditions performed in A. Error bars represent means ± SEM, (n=3). Statistical significance was measured by two-tailed unpaired *t*-test (** $p<0.01$). (C) Htt-Q74 expressing cells were cultivated at 37ºC (C) in the absence (-Dox) or presence of Doxycycline (+Dox) for 3 days, treated with 5 μM AZC and immunoblots probed with the indicated antibodies. (D) Htt-Q74 expressing cells were cultivated as described in (A) and protein extraction was performed in 8 M Urea or (E) Acetone precipitation and TCA solubilization. (F) HSF1 mRNA levels determined by qRT-PCR from Htt-Q74 expressing cells under control (C) and Heat shock conditions (HS). The value given for mRNA levels in the control sample (C, -Dox) was set at 1. Error bars represent means ± SEM, (n=3). (G) Htt-Q74 expressing cells were cultivated in the absence or presence of Dox for 3 days and exposed to a 1 h heat shock at 42ºC and samples collected along the recovery period at 37ºC over 7 h. (H) The inducible PC12 cell line expressing the androgen receptor (AR) fused to a Q10 (non-pathogenic) or Q112 (pathogenic) repeat were cultivated at 37ºC (C) in the absence (-Dox) or presence of Doxycycline (+Dox) for 3 days and heat shocked at 1 h at 42ºC, followed by a recovery period at 37ºC for 7 h (HS). (I) Image quantification data of IHC experiments shown in **Figure 1G.** Left panel shows the % of HSF1 positive cells normalized to the total number of cells stained by DAPI in the WT and KIQ175 mice at 12 months of age in the dorsal striatum. Right panel shows the ratio between HSF1 positive cells and HSF1-S303-P stained cells. Data was normalized to WT levels set as 1 for n=3 independent experiments. Error bars represent means ± SEM. Two-tailed unpaired *t*-test (*p-value < 0.05). (J) Wild type C57BL/6 and KIQ175 mice were sacrificed at 2, 6 and 12 months and cortex samples analyzed by immunoblotting for the indicated proteins. See Supplementary Fig. 13 for uncropped immunoblots.

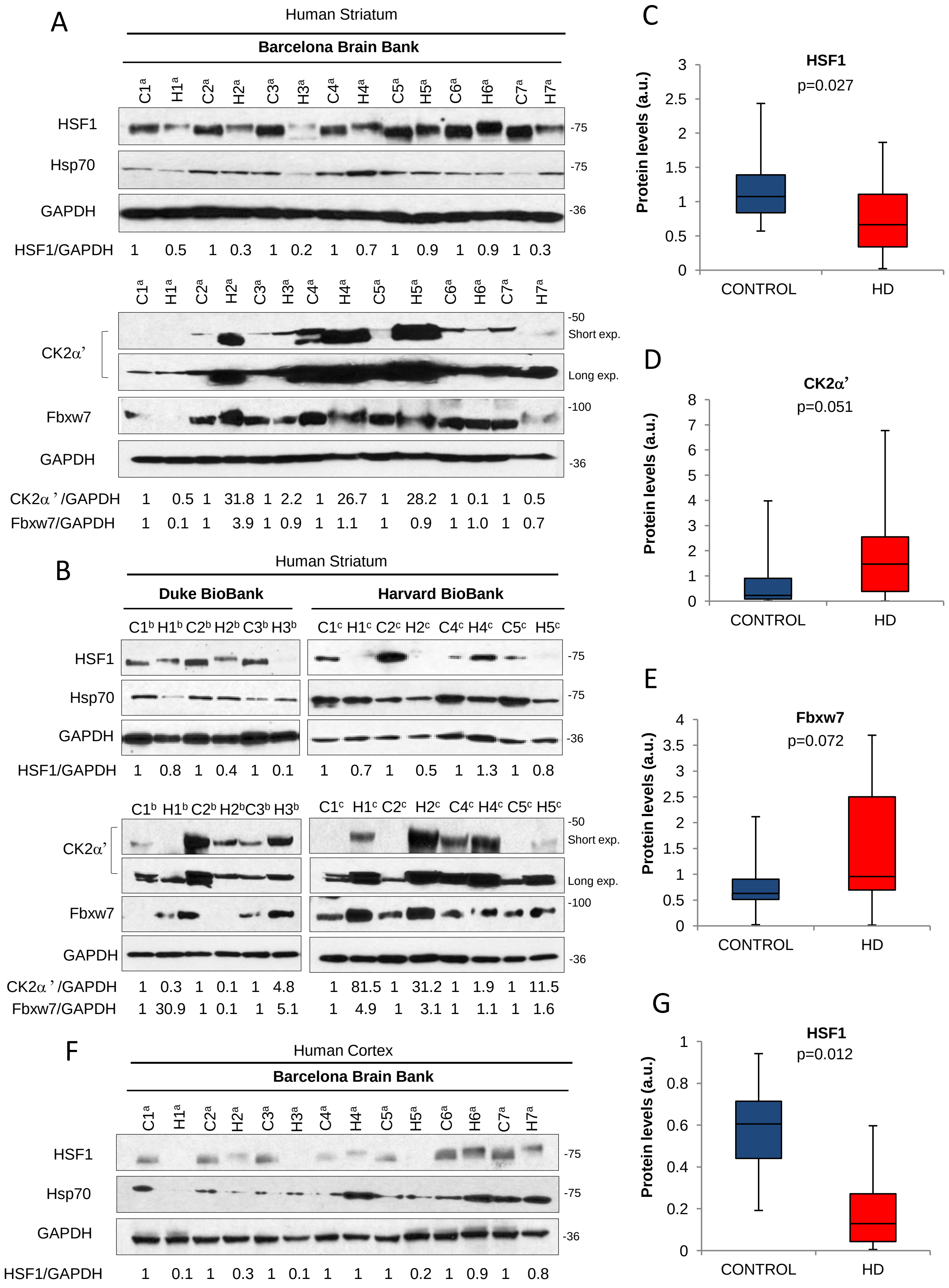
A
Human Striatum
Barcelona Brain Bank
C1a H1a C2a H2a C3a H3a C4a H4a C5a H5a C6a H6a C7a H7a
HSF1
Hsp70
GAPDH
-75
-75
-36
HSF1/GAPDH 1 0.5 1 0.3 1 0.2 1 0.7 1 0.9 1 0.9 1 0.3
CK2α'
Fbxw7
GAPDH
-50
Short exp.
Long exp.
-100
-36
CK2α'/GAPDH 1 0.5 1 31.8 1 2.2 1 26.7 1 28.2 1 0.1 1 0.5
Fbxw7/GAPDH 1 0.1 1 3.9 1 0.9 1 1.1 1 0.9 1 1.0 1 0.7
B
Human Striatum
Duke BioBank
Harvard BioBank
C1b H1b C2b H2b C3b H3b
C1c H1c C2c H2c C4c H4c C5c H5c
HSF1
Hsp70
GAPDH
HSF1/GAPDH 1 0.8 1 0.4 1 0.1 1 0.7 1 0.5 1 1.3 1 0.8
CK2α'
Fbxw7
GAPDH
CK2α'/GAPDH 1 0.3 1 0.1 1 4.8 1 81.5 1 31.2 1 1.9 1 11.5
Fbxw7/GAPDH 1 30.9 1 0.1 1 5.1 1 4.9 1 3.1 1 1.1 1 1.6
F
Human Cortex
Barcelona Brain Bank
C1a H1a C2a H2a C3a H3a C4a H4a C5a H5a C6a H6a C7a H7a
HSF1
Hsp70
GAPDH
HSF1/GAPDH 1 0.1 1 0.3 1 0.1 1 1 1 0.2 1 0.9 1 0.8
C
HSF1
p=0.027
Protein levels (a.u.)
CONTROL
HD
D
CK2α'
p=0.051
Protein levels (a.u.)
CONTROL
HD
E
Fbxw7
p=0.072
Protein levels (a.u.)
CONTROL
HD
G
HSF1
p=0.012
Protein levels (a.u.)
CONTROL
HD

Supplementary Figure 2. HSF1, CK2α' and Fbxw7 protein levels in patients with HD. Related to Figure 1. (A, B) Striatal samples from patients with HD and sex- and age-matched controls from the Barcelona Brain Bank, Duke BioBank and Harvard BioBank were analyzed by immunoblotting. HSF1, CK2α' and Fbxw7 bands from immunoblots were quantified using Image J software (BioRad) and protein values were normalized using GAPDH as loading control and referenced to the corresponding age-sex matched control patient set to 1. (C-E) Variation of HSF1, CK2α' and Fbxw7 protein levels in Control and patients with HD. Values were quantified using Image J software from immunoblots on A and B. Error bars represent ± SD, (n=14 for each group). P values were analyzed by two-tailed unpaired t-test. (E) Cortex from HD patients and sex- and age- matched controls from the Barcelona Brain Bank were analyzed by immunoblotting (**Supplementary Table 1**). (F) Variation of HSF1 protein levels in Control and patients with HD in the Cortex. Values were quantified using Image J software from immunoblots on E. Error bars represent ± SD, (n=7 for each group). P value was analyzed by two-tailed unpaired t-test. See Supplementary Fig. 14 for uncropped immunoblots.

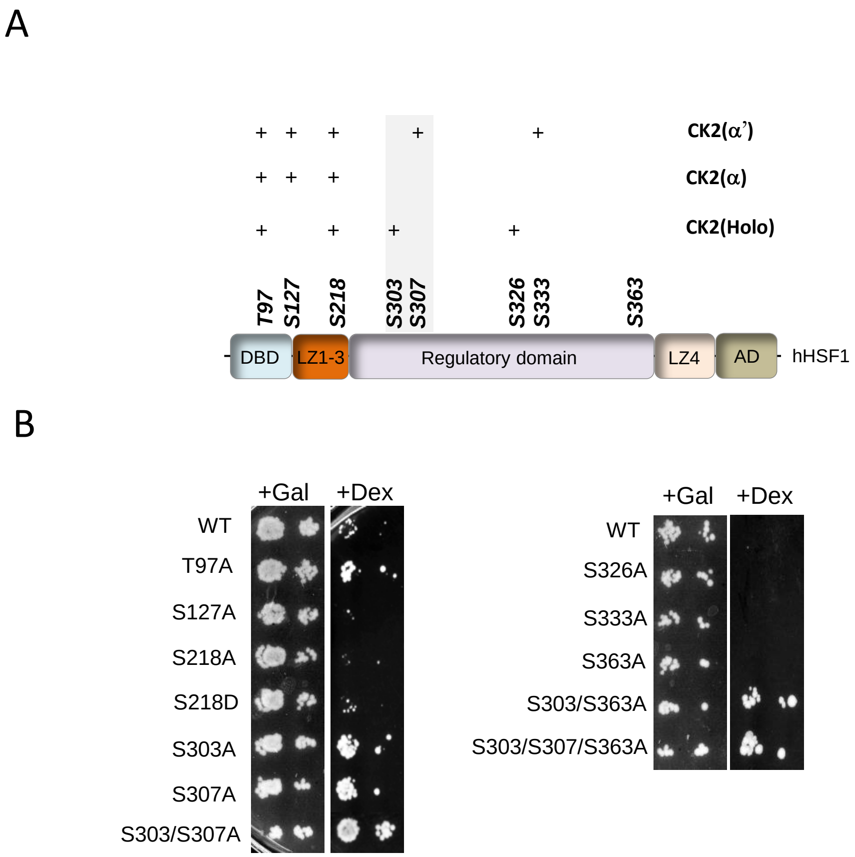

Supplementary Figure 3. CK2-dependent HSF1 phosphorylation site mutagenesis. Related to Figure 3. (A) Diagram of specific HSF1 serine and threonine residues phosphorylated by CK2 *in vitro* by either purified recombinant CK2α, CK2α' or commercial holo-enzyme and evaluated in the humanized HSF1 yeast assay. (B) Yeast strain PS145 transformed with WT human HSF1 plasmid (pRS424-GPD-hHSF1) or the indicated HSF1 mutations corresponding to CK2-phophorylated residues from (A). Cells were plated on either galactose or dextrose supplemented SC medium, plates incubated for 3 days at 30ºC and photographed.

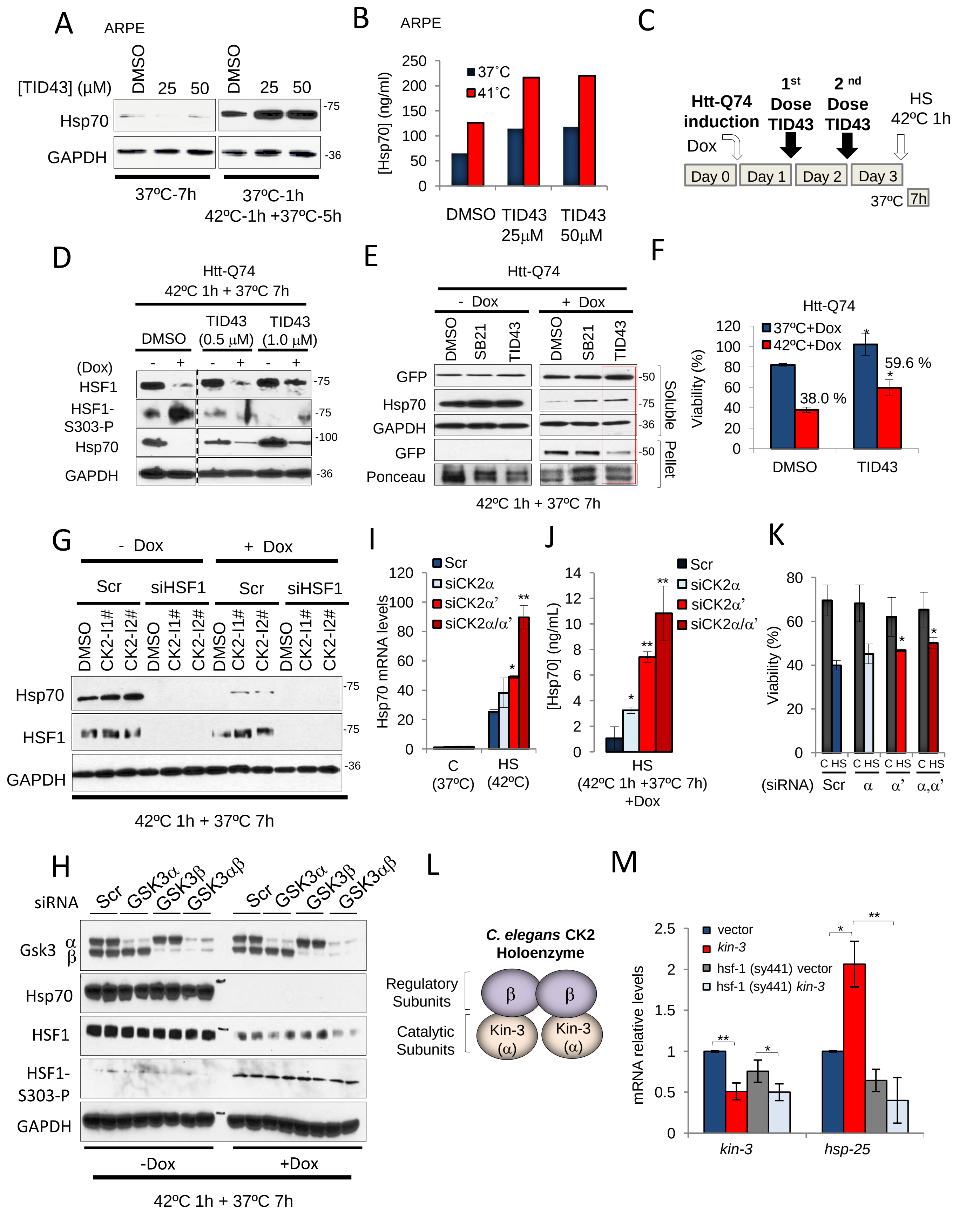

A
ARPE
[TID43] (μM)
DMSO 25 50 DMSO 25 50
Hsp70
GAPDH
37ºC-7h
37ºC-1h
42ºC-1h +37ºC-5h
B
ARPE
[Hsp70] (ng/ml)
37˚C
41˚C
DMSO
TID43 25μM
TID43 50μM
C
Htt-Q74 induction
Dox
1st Dose TID43
2 nd Dose TID43
HS 42ºC 1h
Day 0
Day 1
Day 2
Day 3
37ºC 7h
D
Htt-Q74
42ºC 1h + 37ºC 7h
DMSO
TID43 (0.5 μM)
TID43 (1.0 μM)
(Dox)
HSF1
HSF1-S303-P
Hsp70
GAPDH
E
Htt-Q74
- Dox
+ Dox
DMSO SB21 TID43
GFP
Hsp70
GAPDH
Ponceau
Soluble
Pellet
42ºC 1h + 37ºC 7h
F
Htt-Q74
Viability (%)
37ºC+Dox
42ºC+Dox
38.0 %
59.6 %
DMSO
TID43
G
- Dox
+ Dox
Scr
siHSF1
DMSO CK2-I1# CK2-I2#
Hsp70
HSF1
GAPDH
42ºC 1h + 37ºC 7h
I
Hsp70 mRNA levels
Scr
siCK2α
siCK2α'
siCK2α/α'
C (37ºC)
HS (42ºC)
J
[Hsp70] (ng/mL)
HS (42ºC 1h +37ºC 7h) +Dox
K
Viability (%)
C HS
(siRNA) Scr α α' α,α'
H
siRNA
Scr GSK3α GSK3β GSK3αβ
Gsk3 α β
Hsp70
HSF1
HSF1-S303-P
GAPDH
-Dox
+Dox
42ºC 1h + 37ºC 7h
L
C. elegans CK2 Holoenzyme
Regulatory Subunits
Catalytic Subunits
β
Kin-3 (α)
M
mRNA relative levels
vector
kin-3
hsf-1 (sy441) vector
hsf-1 (sy441) kin-3
kin-3
hsp-25

Supplementary Figure 4. CK2 pharmacological inhibition increases HSF1 protein activity and stability. Related to Figure 4. (A) The human retinal pigment epithelial cell line ARPE was treated for 1 h with 25 μm or 50 μm TID43 at 37ºC, heat shocked for 1 h at 41ºC, recovered at 37ºC for 16 h and immunoblotted for Hsp70 and GAPDH. (B) Hsp70 protein levels were measured by ELISA under the same the experimental conditions as in (A). (C) Experimental design for the addition of TID43 consisting of two doses (24 h and 48 h) after Htt-Q74 induction by Dox. (D) Htt-Q74 cells were induced with Dox (+) or not (-) treated with 0.5 μM and 1 μM TID43 as described in (C) and protein extracts subjected to immunoblotting. Samples are from the same membrane and same exposure and immunoblots were cropped to show relevant data. (E) Htt-Q74 expressing cells were treated as described in (C) using 1 μM SB216763 or TID43. Cells were lysed and soluble and pellet fractions assayed by immunoblotting with the indicated antibodies. GFP in the pellet fraction was used as a marker for Htt-Q74-GFP aggregation. Samples are on the same membrane at the same exposure and immunoblots were cropped to show relevant data. (F) Htt-Q74 cell viability was analyzed in the presence of Dox comparing DMSO control and cells treated with 1 μM TID43 upon heat shock conditions as described in (D). Data is presented as percentage of viable cells compared with untreated cells in the absence of Dox (100%). Error bars represent means ± SEM, (n=3). Statistical significance was measured by two-tailed unpaired *t*-test comparing the TID43 group to DMSO group (* $p<0.05$, ** $p<0.01$). (G) PC12-Htt-Q74 expressing cells were transfected with siRNA against HSF1 or non-targeted siRNA (Scr) for 48h. Cells were treated with two different CK2 inhibitors (CK2-I1# for SB21 and CK2-I2# for TID43) at 1mM for 6h. Cells were heat shocked at 42ºC for 1h and allowed to recover at 37ºC for 7h. Protein samples were collected and subjected to immunoblotting for the indicated proteins. (H) Htt-Q74 cells were transfected with siRNA against Gsk3α or Gsk3β separately or together using scrambled siRNA (Scr) as control. After 24 h cells were incubated at 37ºC (C) in the absence (-Dox) or presence (+Dox) of doxycycline for 2 days, heat shocked at 42ºC for 1 h, recovered at 37ºC for 7 h and immunoblotted for the indicated proteins. (I) Hsp70 mRNA levels quantitated by qRT-PCR after transfection with the indicated siRNA or scrambled control (Scr). Error bars represent means ± SEM, (n=3). Statistical significance was measured comparing the siRNA group to Scr group under HS conditions by two-tailed unpaired *t*-test (* $p<0.05$, ** $p<0.01$). (J) Hsp70 protein levels by ELISA (Enzo) under different siRNA conditions as in (I). Statistical significance was measured comparing siRNA groups to the Scr group. (K) Viability of cells expressing Htt-Q74 and transfected with siRNA against CK2α or CK2α' in the presence of Dox was analyzed using Cell Titer Glo comparing Control (Gray bars) and Heat Shocked (Colored bars). Data is presented as percentage of viable cells compared to untreated Scr cells. Statistical significance was measured comparing siRNA groups to the Scr group under HS conditions. Error bars represent means ± SEM, (n=3). Two-tailed unpaired *t*-test (* $p<0.05$, ** $p<0.01$). (L) Diagram of the *C. elegans* CK2 holoenzyme. (M) *C. elegans* pathogenic Q37 AM470 strain (Q37::YFP) and the HD model strain Q37::YFP;*hsf-1*(sy441) hypo-morphic mutant [61] were treated with empty vector or *E. coli* expressing RNAi against *kin-3* (CK2a ortholog) and qRT-PCR analysis conducted for *kin-3* and *hsp-25* transcript abundance. Data are relative to the levels of each transcript in the control worm Q37::YFP + empty vector. Error bars represent means ± SEM, (n=3). Two-tailed unpaired *t*-test (* $p<0.05$, ** $p<0.01$). See Supplementary Fig. 15 for uncropped immunoblots.

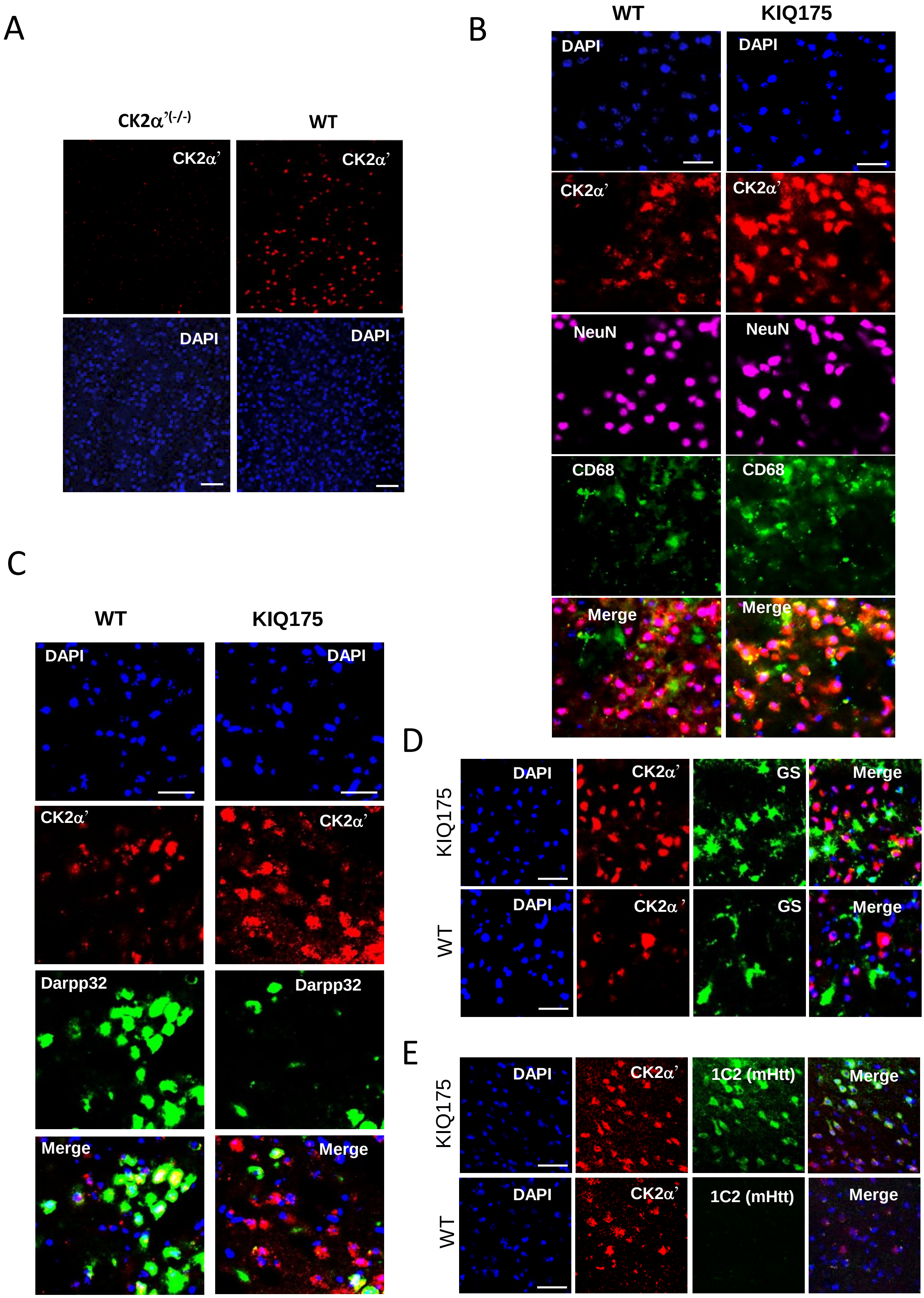
A
CK2α'(-/-)
WT
CK2α'
DAPI
B
WT
KIQ175
DAPI
CK2α'
NeuN
CD68
Merge
C
WT
KIQ175
DAPI
CK2α'
Darpp32
Merge
D
KIQ175
WT
DAPI
CK2α'
GS
Merge
E
KIQ175
WT
DAPI
CK2α'
1C2 (mHtt)
Merge

Supplementary Figure 5. CK2α' cellular sub-type expression in the dorsal striatum of KIQ175. Related to Figure 5. (A) Coronal section of the striatum of WT and CK2α´(-/-) mice at 3 months of age showing specific staining of CK2α' only in WT mice. (B-E) Coronal section of the striatum of WT and KIQ175 mice at 6 months of age, showing staining of (B) CK2α'(red) and the neuronal marker NeuN (magenta) co-localize but there is no co-localization with the reactive microglia marker CD68 (green). (C) Co-localization of CK2α'(red) and the Darpp32 (green) MSN marker. (D) CK2α'(red) and GS, astrocytes (green) do not co-localize. (E) CK2α' (red) and mHtt (green) co-localize in the KIQ175 mice. Nuclei were detected with DAPI. Scale bar: A 40μm, B-E 10μm.

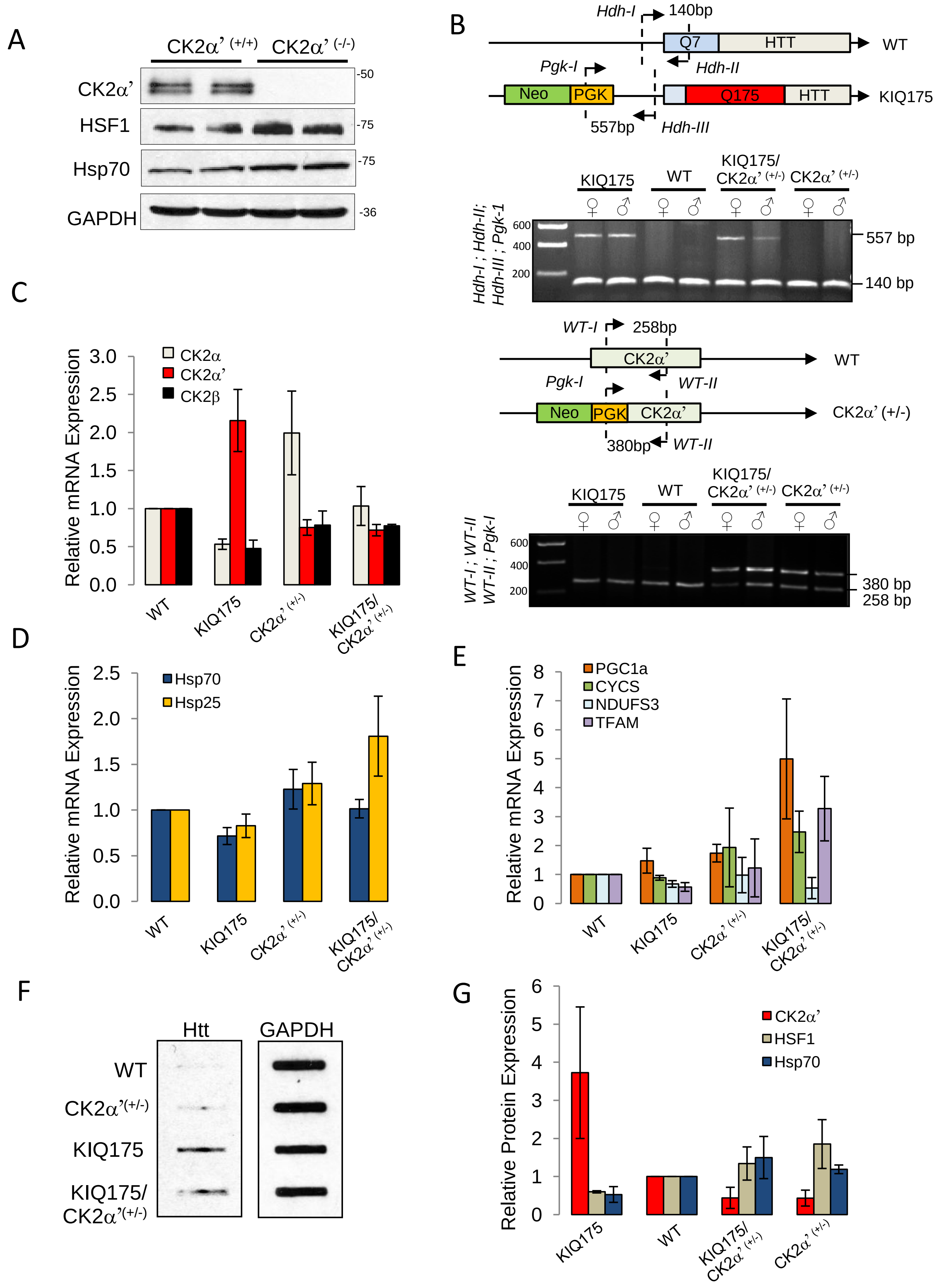

A
CK2α’ (+/+)
CK2α’ (-/-)
CK2α’
HSF1
Hsp70
GAPDH
-50
-75
-75
-36
B
Hdh-I
140bp
Q7
HTT
WT
Pgk-I
Hdh-II
Neo
PGK
Q175
HTT
KIQ175
557bp
Hdh-III
KIQ175
WT
KIQ175/ CK2α’ (+/-)
CK2α’ (+/-)
Hdh-I ; Hdh-II; Hdh-III ; Pgk-1
600
400
200
557 bp
140 bp
WT-I
258bp
CK2α’
WT
Pgk-I
WT-II
Neo
PGK
CK2α’
CK2α’ (+/-)
380bp
WT-II
WT-I ; WT-II WT-II ; Pgk-I
380 bp
258 bp
C
Relative mRNA Expression
CK2α
CK2α’
CK2β
WT
KIQ175
CK2α’ (+/-)
KIQ175/ CK2α’ (+/-)
D
Hsp70
Hsp25
E
PGC1a
CYCS
NDUFS3
TFAM
F
Htt
GAPDH
WT
CK2α’(+/-)
KIQ175
KIQ175/ CK2α’(+/-)
G
Relative Protein Expression
CK2α’
HSF1
Hsp70

Supplementary Figure 6. **CK2α' Hetorozygous mice increase HSF1 transcriptional activity and decrease mHtt aggregates.** Related to Figure 7. (A) Wild type C57BL/6 (CK2α' $^{+/+}$) and homozygous CK2α' knock-out mice (CK2α' $^{-/-}$) mice were sacrificed at 3 months and protein extracts from the striatum subjected to immunoblotting with the indicated antibodies. (B) Diagram of primer design for mouse genotyping of the Htt and CK2α' loci showing PCR genotyping of one representative male and one female KIQ175, WT (CK2α' $^{+/+}$), KIQ175/(CK2α' $^{+/-}$), and CK2α' $^{+/-}$ mouse per genotype. (C-E) qRT-PCR analysis for WT, KIQ175, CK2α' (+/-) and KIQ175/ CK2α' (+/-) mice was conducted from striatal mRNA at 6 months of age for: (C) Casein kinase 2 catalytic (α and α') and regulatory subunits (β), (D) Hsp70 and Hsp25 and (E) Mitochondrial activity-related genes PGC1a and its downstream targets CYCs, NDUFS3 and TFAM. All data was normalized to GAPDH expression and to WT expression levels set as 1. Error bars represent means ± SEM, (n=3 animals). (F) Filter retardation assay for Htt aggregation from striatal tissue of 6 month old KIQ175, WT (CK2α' $^{+/+)}$, KIQ175/(CK2α' $^{+/-}$), and CK2α' $^{+/-}$ mice. (G) Image quantification for CK2α', HSF1 and Hsp70 protein levels in the striatum of WT, KIQ175, CK2α' (+/-) and KIQ175/ CK2α' (+/-) mice at 6 months of age (n=3). See Supplementary Fig. 15 for uncropped immunoblots.

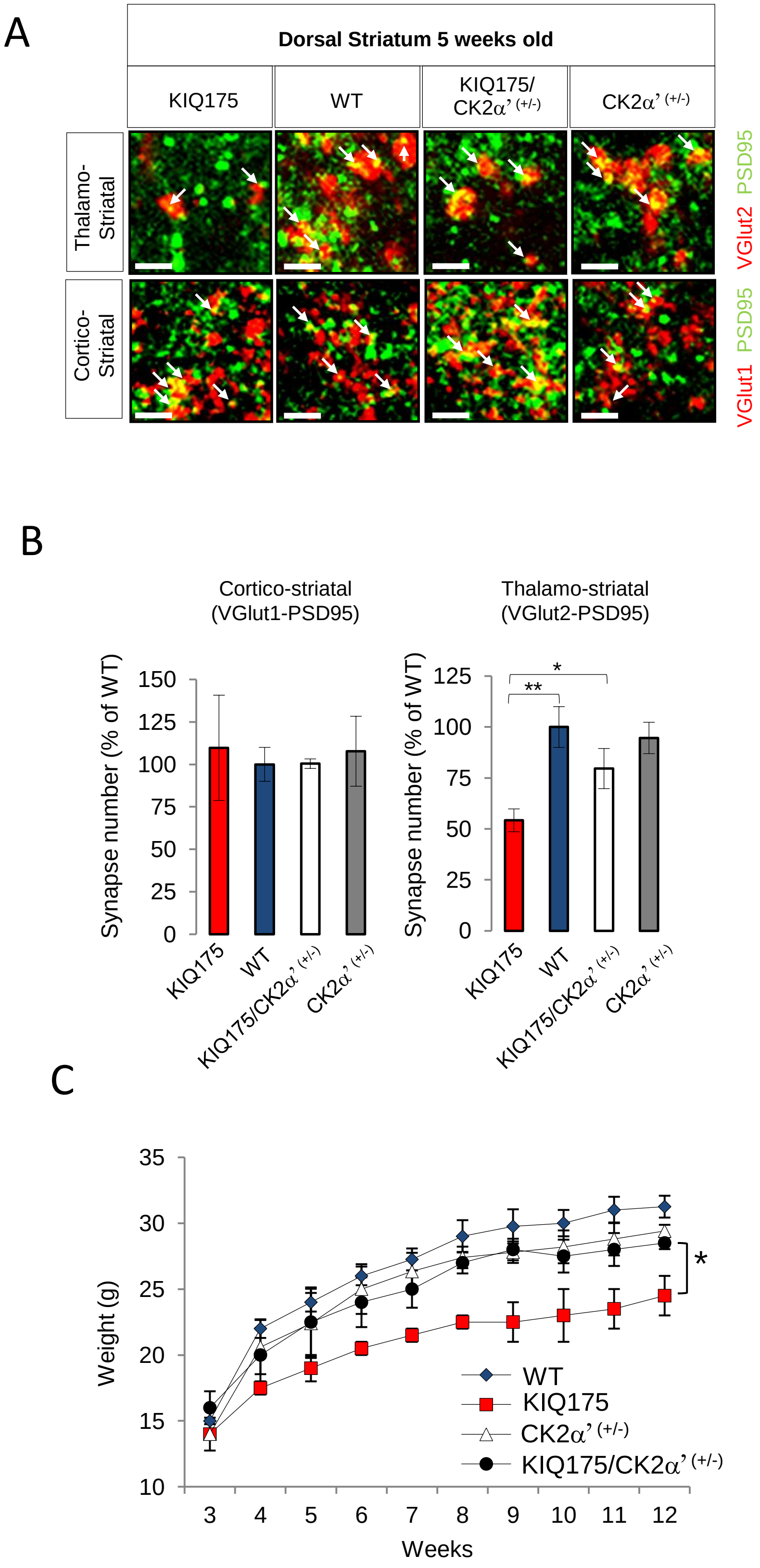
A
Dorsal Striatum 5 weeks old
KIQ175
WT
KIQ175/ CK2α' (+/-)
CK2α' (+/-)
Thalamo-Striatal
Cortico-Striatal
VGlut2 PSD95
VGlut1 PSD95
B
Cortico-striatal (VGlut1-PSD95)
Thalamo-striatal (VGlut2-PSD95)
Synapse number (% of WT)
150
125
100
75
50
25
0
*
**
KIQ175
WT
KIQ175/CK2α' (+/-)
CK2α' (+/-)
C
Weight (g)
35
30
25
20
15
10
3
4
5
6
7
8
9
10
11
12
Weeks
WT
KIQ175
CK2α' (+/-)
KIQ175/CK2α' (+/-)
*

Supplementary Figure 7. **CK2α´ heterozygosity increases synapse number and body weight at early onset in the KIQ175.** Related to Figure 8. (A) Images from co-localization (synapse) of the cortico-striatal pre-synaptic marker (VGlut1), the thalamo-striatal pre-synaptic marker (VGlut2) and the post-synaptic marker PSD95 in the dorsal striatum of KIQ175, WT (CK2α´$^{+/+)}$, KIQ175/(CK2α´$^{+/-)}$, and CK2α´$^{+/-}$ mice at 5 weeks. Synapses are indicated with white arrows. Sale bar, 10 μM. (B) Quantification of VGlut1-PSD95 and VGlut2-PSD95 co-localized synaptic puncta from (A). Error bars indicate mean ± SEM, (n=3 animals per genotype, 3 sections per animal, 15 sections per scan). Unpaired t-test * p<0.05, ** p<0.01. (C) Body weight longitudinal study for WT (n=4), KIQ175 (n=3), CK2α´ (+/-) (n=5) and KIQ175/ CK2α´ (+/-) (n=3) over 9 weeks following weaning, represented in grams (g). Only males were used in this study to minimize weight differences. Error bars indicate mean ± SEM, Unpaired *t*-test * p<0.05.

Fig. 1B

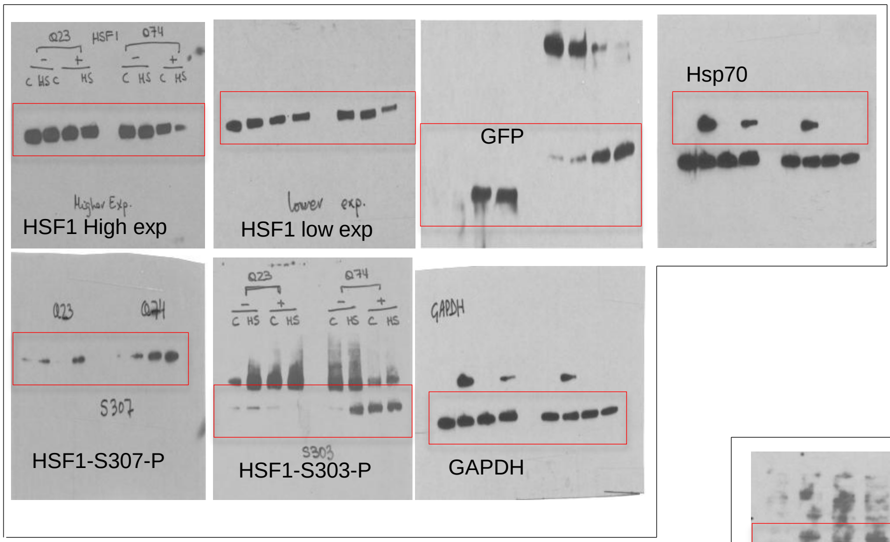

Fig. 1E

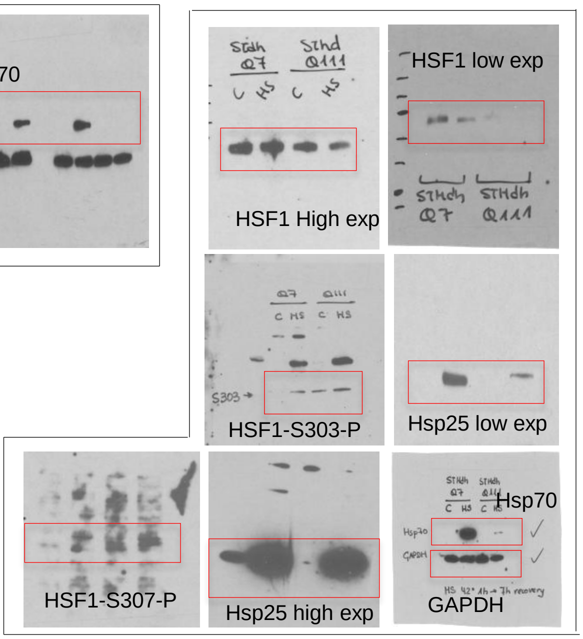

Fig. 1F

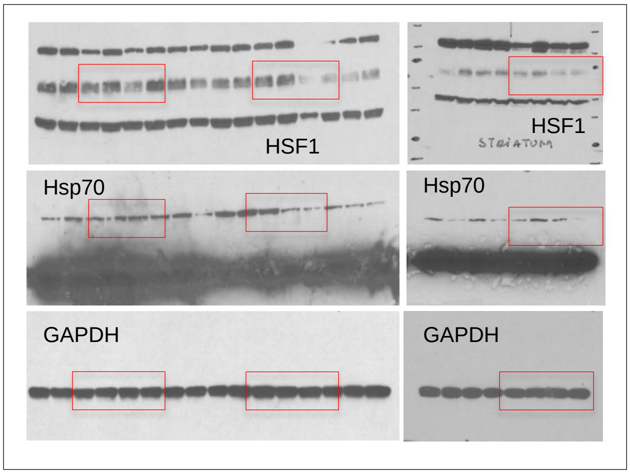

Fig. 1H

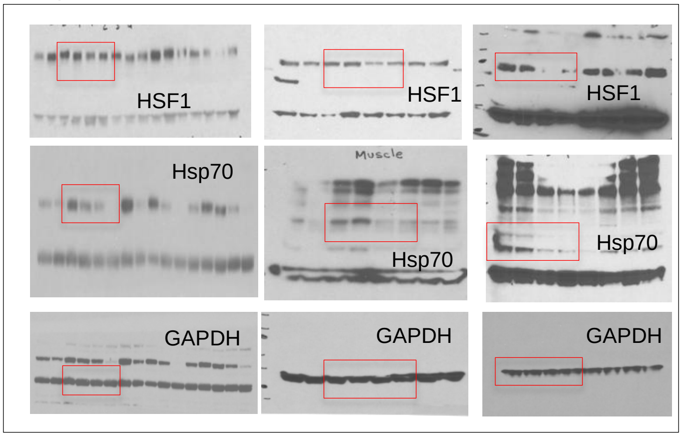

Fig. 1I

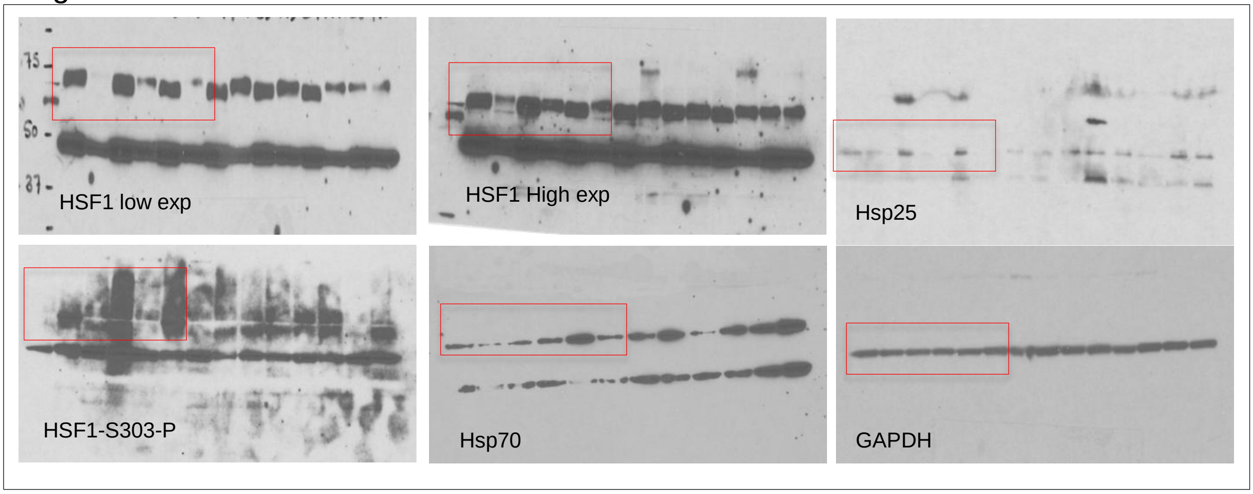

Supplementary Figure 8. Uncropped images for immunoblots for Fig. 1. Red boxes show approximate image used for presentation.

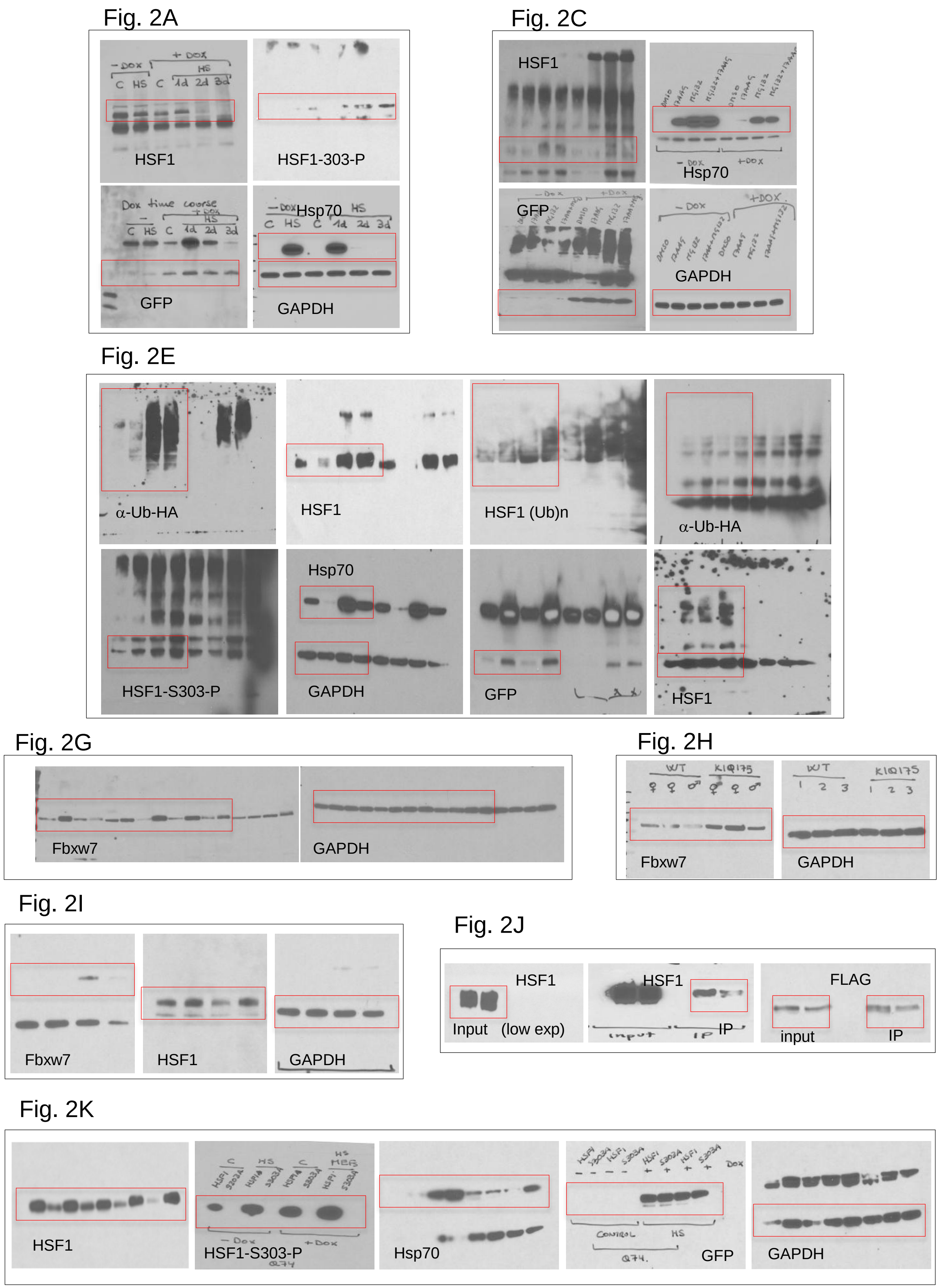

Supplementary Figure 9. Uncropped images for immunoblots for Fig. 2. Red boxes show approximate image used for presentation.

Fig. 4B

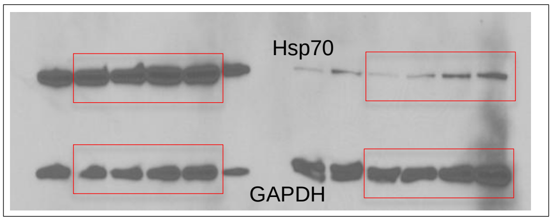

Fig. 4C

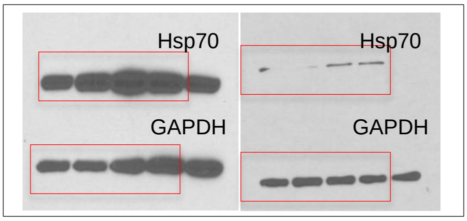

Fig. 4D

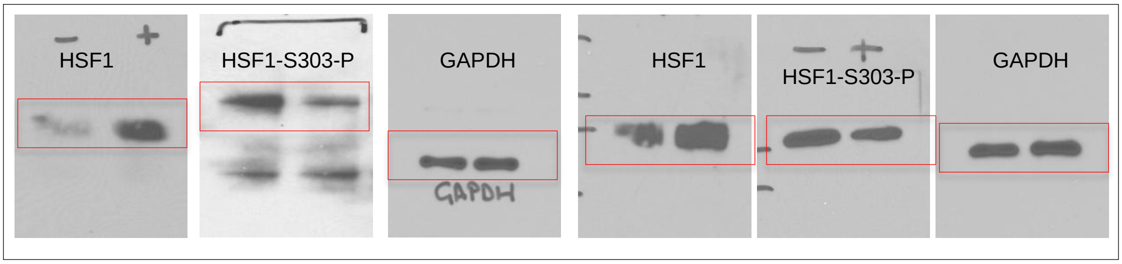

Fig. 4H

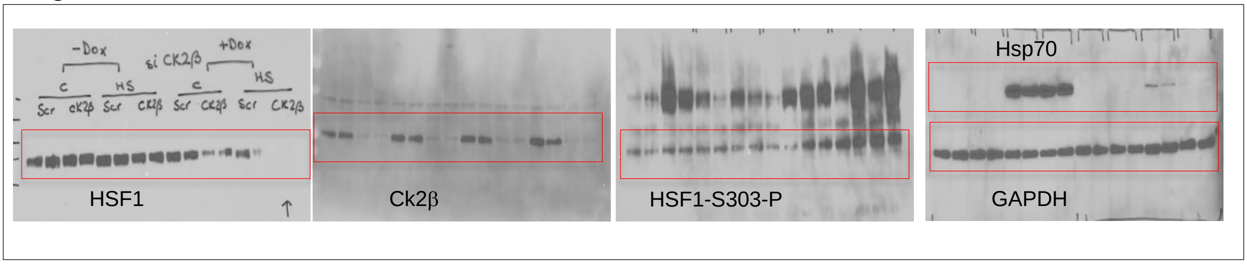

Fig. 4I

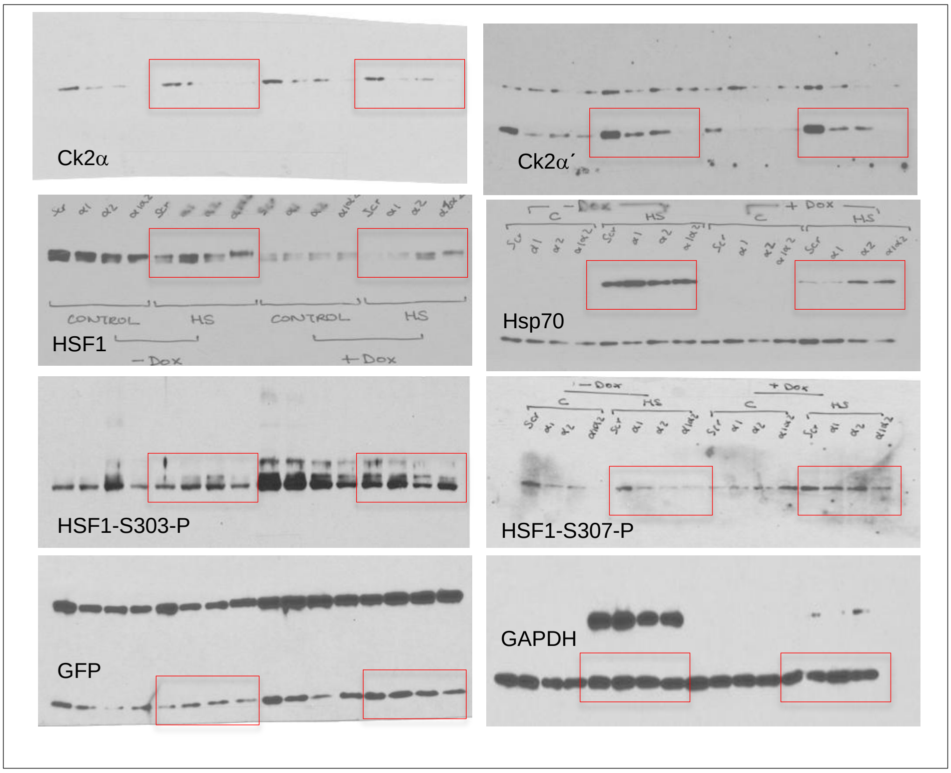

Supplementary Figure 10. Uncropped images for immunoblots for Fig. 4. Red boxes show approximate image used for presentation.

Fig. 5A

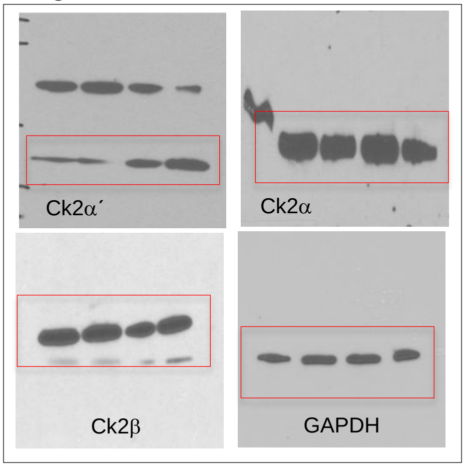

Fig. 5C

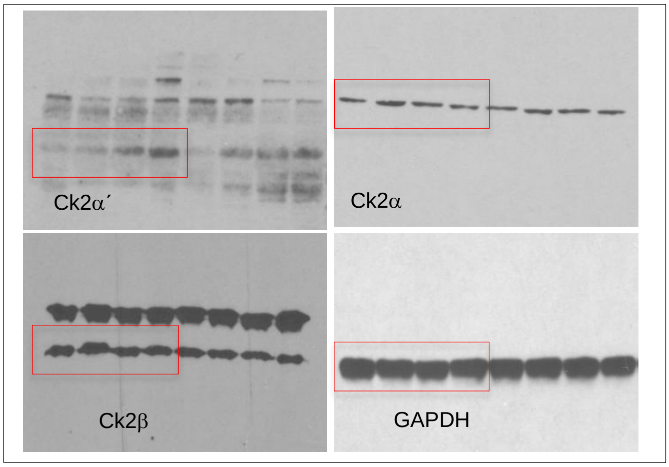

Fig. 5D

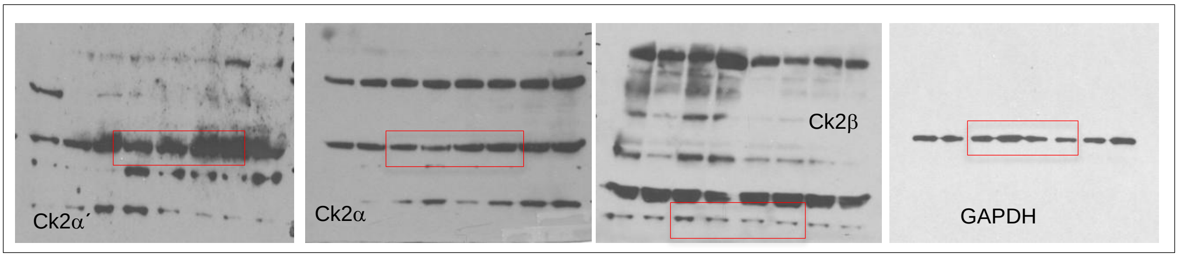

Fig. 5G

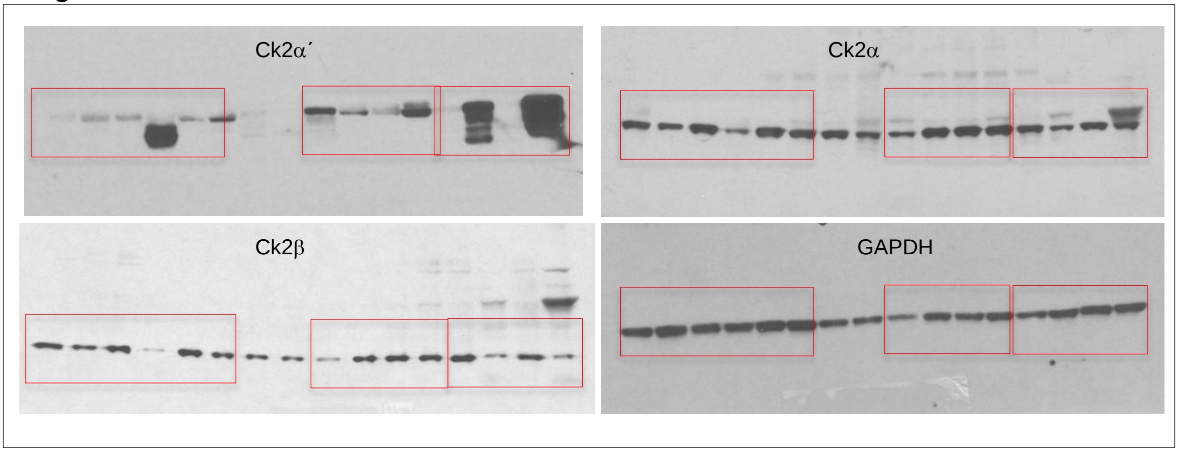

Supplementary Figure 11. Uncropped images for immunoblots for Fig. 5. Red boxes show approximate image used for presentation.

Fig. 6A

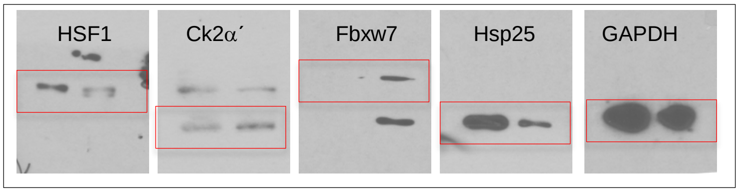

Fig. 6B

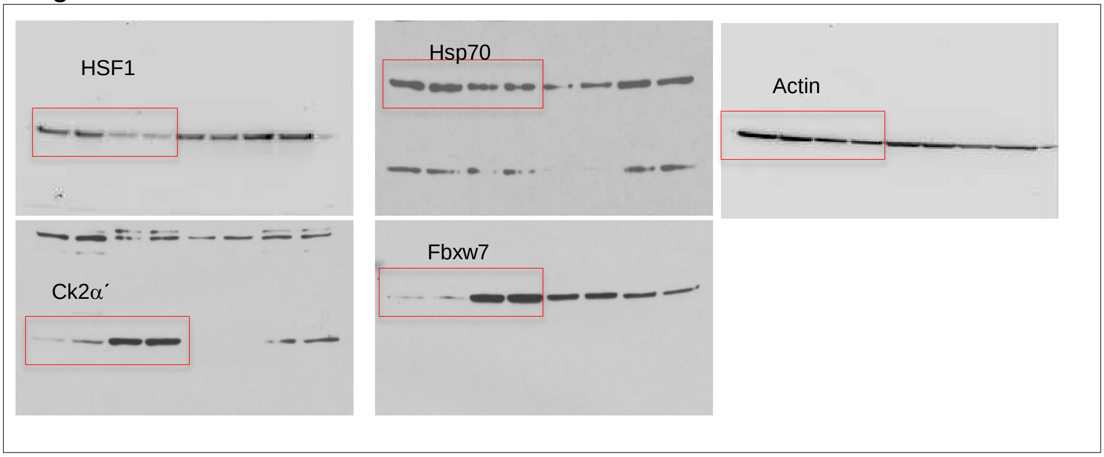

Fig. 7E

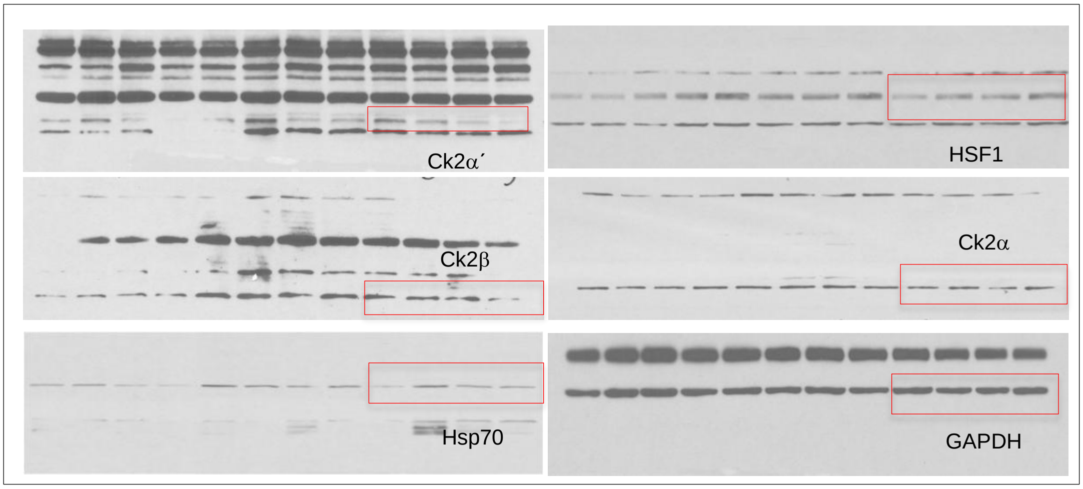

Fig. 7F

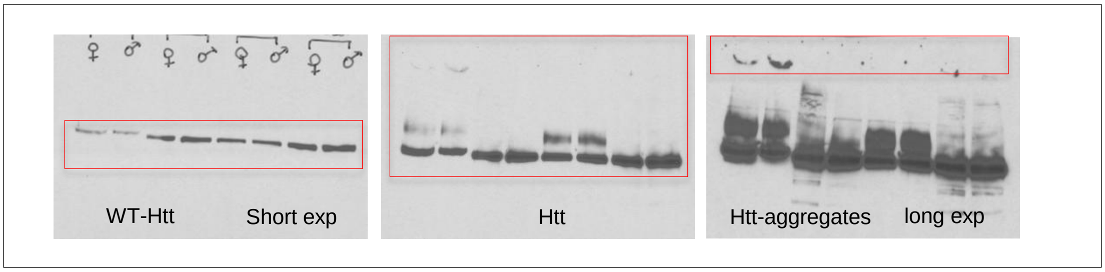

Supplementary Figure 12. Uncropped images for immunoblots for Fig. 6 and Fig. 7. Red boxes show approximate image used for presentation.

Supplementary Fig. 1A

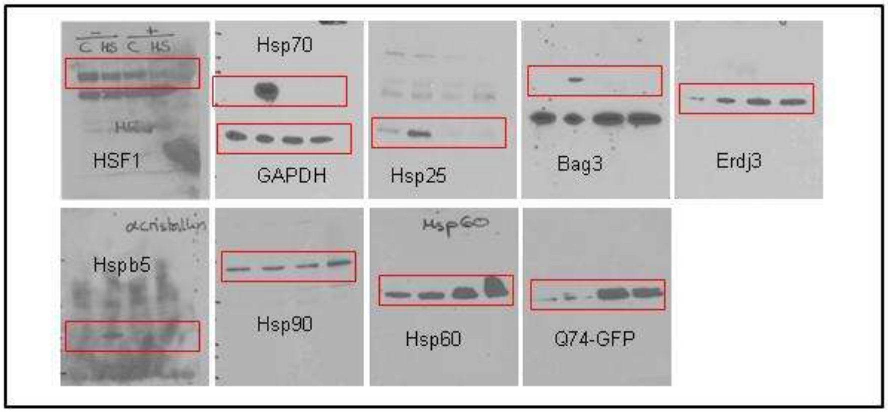

Supplementary Fig. 1D

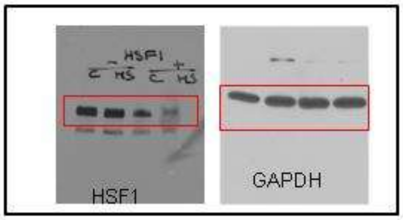

Supplementary Fig. 1G

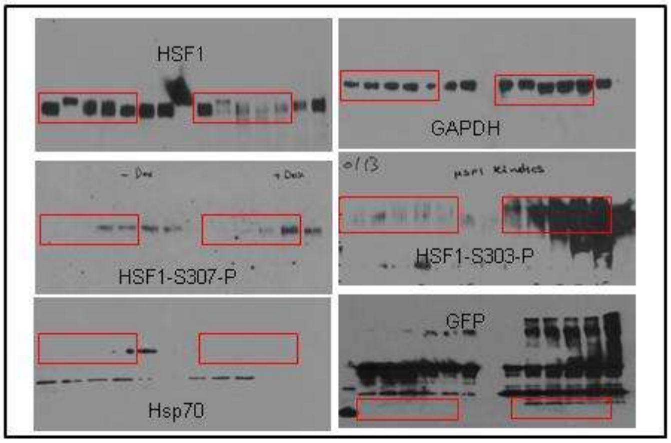

Supplementary Fig. 1E

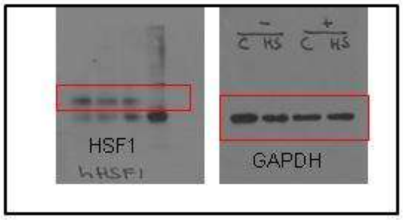

Supplementary Fig. 1H

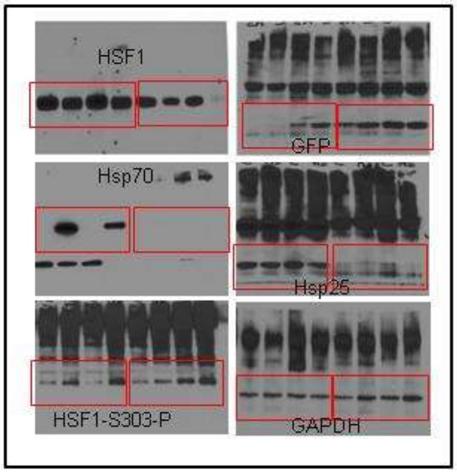

Supplementary Fig. 1J

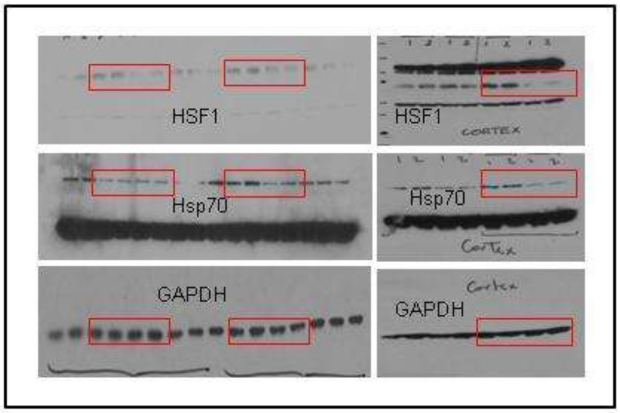

Supplementary Figure 13. Uncropped images for immunoblots for **Supplementary Fig. 1**. Red boxes show approximate image used for presentation.

Supplementary Fig. 2A

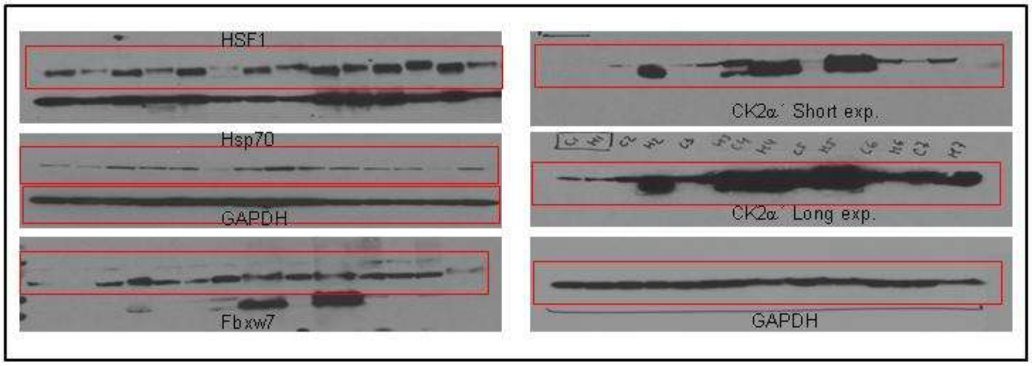

Supplementary Fig. 2B

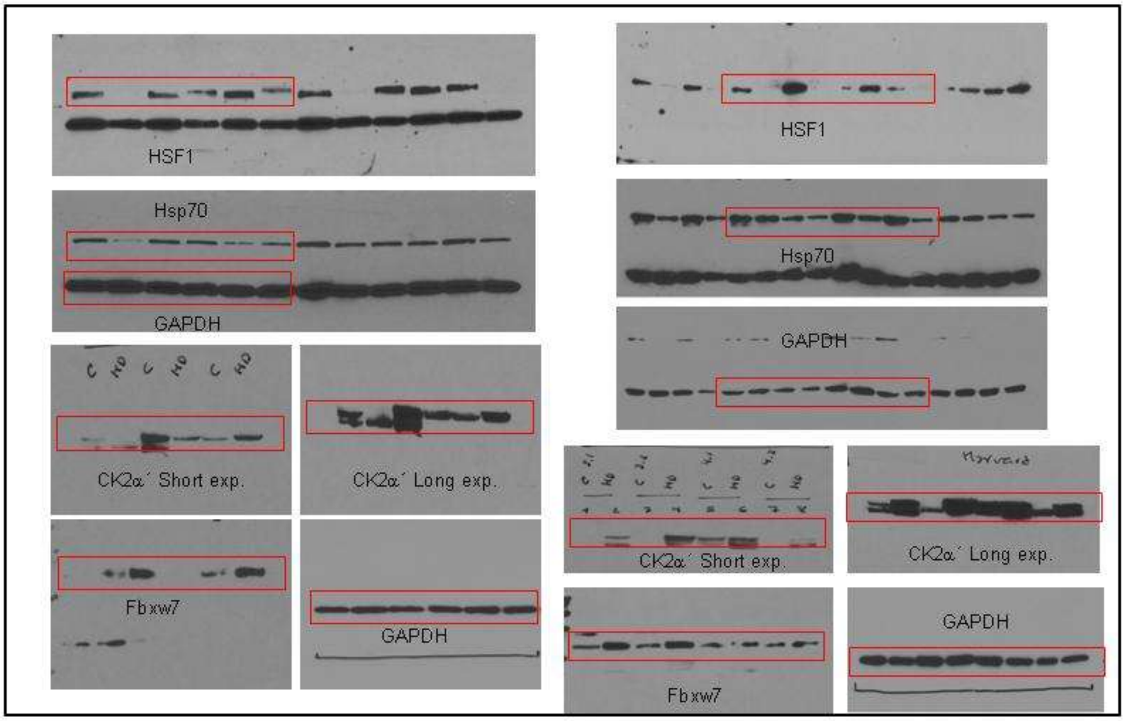

Supplementary Fig. 2C

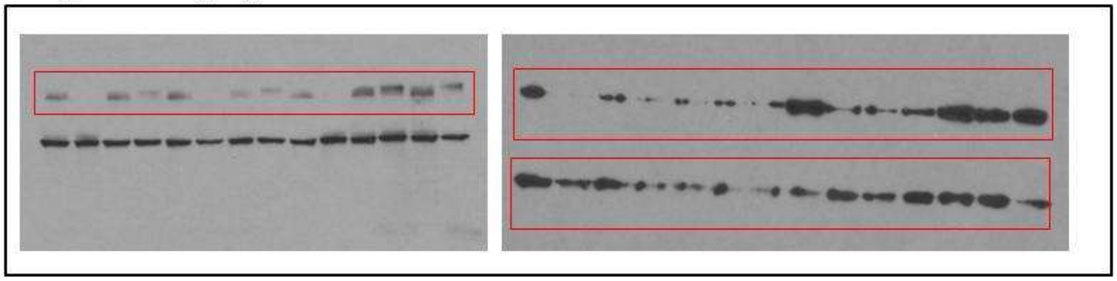

Supplementary Figure 14. Uncropped images for immunoblots for **Supplementary Fig. 2**. Red boxes show approximate image used for presentation.

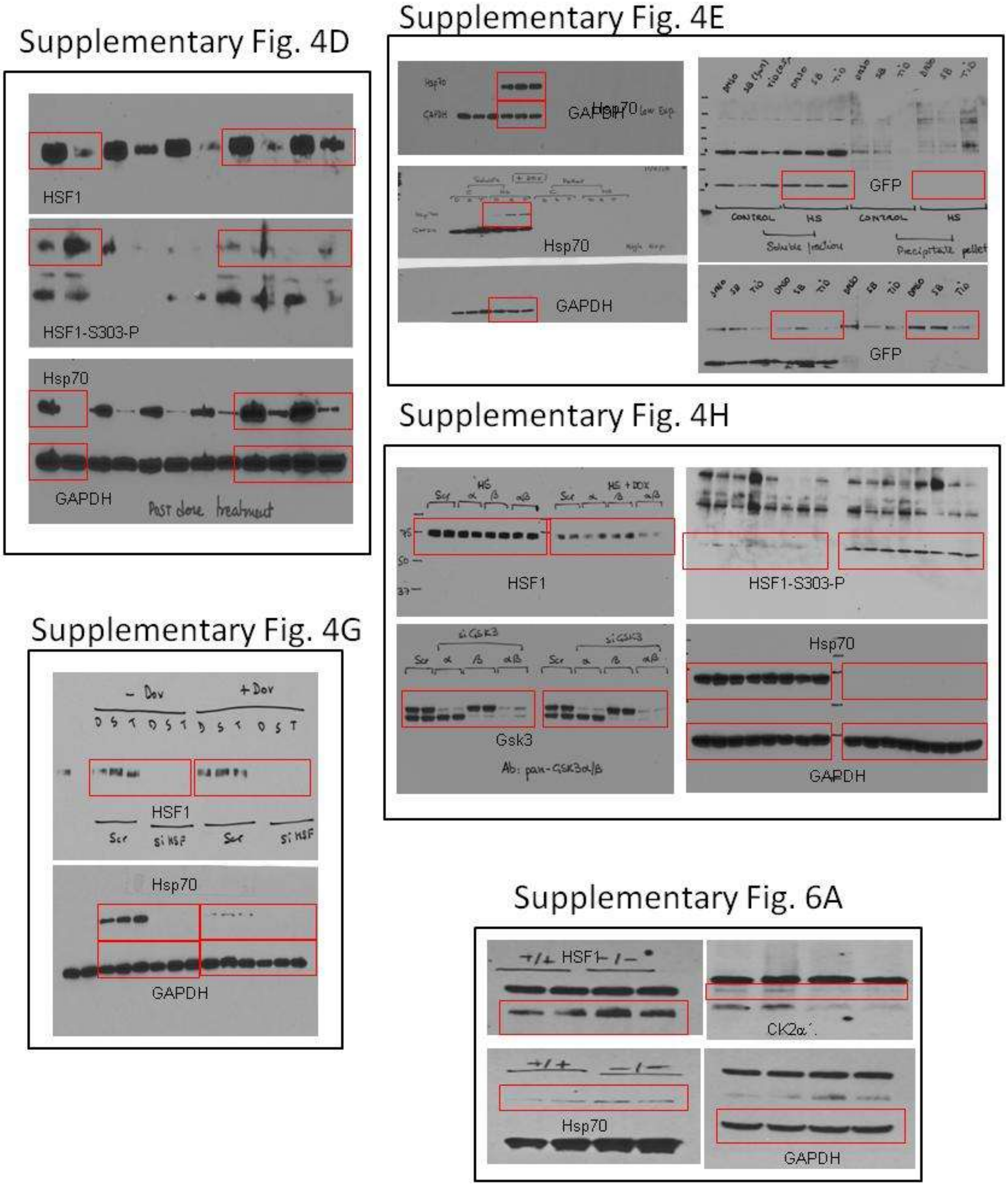

Supplementary Figure 15. Uncropped images for immunoblots for **Supplementary Fig. 4 and Supplementary Fig 6**. Red boxes show approximate image used for presentation.

Table 1. HD Brain Sample description

Sample Code	Age	Sex	Source	PMD	Disease Grade
C1a	73	F	B.B	7.0	-
H1a	72	F	B.B	7.0	Grade 4
C2a	73	F	B.B	5.5	-
H2a	72	F	B.B	5.0	Grade 4
C3a	51	M	B.B	4.0	-
H3a	68	M	B.B	4.0	Grade 4
C4a	70	M	B.B	13.0	-
H4a	71	M	B.B	10.2	Grade 4
C5a	47	F	B.B	9.6	-
H5a	65	F	B.B	15.2	Grade 4
C6a	53	M	B.B	3.0	-
H6a	59	M	B.B	5.5	Grade 4
C7a	46	M	B.B	15.0	-
H7a	60	M	B.B	13.1	Grade 4
C1b	65	F	D.B	13.58	-
H1b	54	F	D.B	18.0	na
C2b	70	M	D.B	13.0	-
H2b	73	M	D.B	4.5	na
C3b	53	M	D.B	3.0	-
H3b	58	M	D.B	1.85	na
C1c	74	M	H.B	21.0	-
H1c	74	M	H.B	19.71	Grade 3
C2c	64	M	H.B	22.78	-
H2c	64	M	H.B	19.00	Grade 3
C4c	62	M	H.B	21.41	-
H4c	61	M	H.B	27.31	Grade 4
C5c	73	F	H.B	26.92	-
H5c	74	F	H.B	26.03	Grade 4

C: Control**, H**: Huntington, **M**; Male, **F**; Female, **B.B**: Barcelona Brain Bank, **D.B**: Duke Kathleen Price Bryan Brain Bank, **H.B**: Harvard Brain Tissue Resource Center, **PMD**: Post-mortem delay (h), **na**: not available.
